# Multi-Layered Cancer Chromosomal Instability Phenotype

**DOI:** 10.3389/fonc.2013.00302

**Published:** 2013-12-11

**Authors:** Anna V. Roschke, Ester Rozenblum

**Affiliations:** ^1^Genetics Branch, Center for Cancer Research, National Cancer Institute, National Institutes of Health, Bethesda, MD, USA

**Keywords:** W-CIN, chromosomal instability, intra-tumor heterogeneity, aneuploidy, cancer

## Abstract

Whole-chromosomal instability (W-CIN) – unequal chromosome distribution during cell division – is a characteristic feature of a majority of cancer cells distinguishing them from their normal counterparts. The precise molecular mechanisms that may cause mis-segregation of chromosomes in tumor cells just recently became more evident. The consequences of W-CIN are numerous and play a critical role in carcinogenesis. W-CIN mediates evolution of cancer cell population under selective pressure and can facilitate the accumulation of genetic changes that promote malignancy. It has both tumor-promoting and tumor-suppressive effects, and their balance could be beneficial or detrimental for carcinogenesis. The characterization of W-CIN as a complex multi-layered adaptive phenotype highlights the intra- and extracellular adaptations to the consequences of genome reshuffling. It also provides a framework for targeting aggressive chromosomally unstable cancers.

## Introduction

Most cancers have an abnormal chromosomal content characterized by clonal changes in chromosomal structure and number. It is now known that greater than 90% of solid tumors and 75% of blood cancers show some degree of genomic disbalances and are aneuploid (1). In addition, cancer cell populations very often show non-clonal cell-to-cell chromosomal heterogeneity. This heterogeneity is a marker of ongoing chromosomal instability in cancers – accelerated rates of changes in chromosome structure, gains and losses of chromosome segments or whole chromosomes. Whole-chromosomal instability (W-CIN) – unequal chromosome distribution during cell division – is a characteristic feature of a majority of cancer cells distinguishing them from their normal counterparts.

Chromosomal instability contributes to transformation by altering the dosage of oncogenes and tumor-suppressors. Gain and loss of chromosomal material in neoplastic cell populations is considered to be a process of diversification that leads to the survival of the fittest clones (2, 3). The evolution of cancer cells from benign tumor to invasive metastasis appears to correlate with increased aneuploidy and karyotypic complexity (4). Both aneuploidy and W-CIN have been associated with poor patient prognosis, metastasis, and resistance to chemotherapeutics (5–14). Thus, understanding the mechanisms that cause W-CIN as well as mechanisms that allow W-CIN cells to survive and acquire malignant features, offers an attractive possibility to interfere with tumor aggressiveness, and enhance the efficiency of cancer therapy.

## Complexity of Cancer Genomes

Cancer genomes are rearranged compared to genomes of normal cells. They display several types of genomic alterations, which include point or oligobase mutations, structural rearrangements (deletions, duplications, insertions, inversions, amplifications, translocations), and whole chromosome copy-number changes (gains and losses of the whole chromosomes).

The complexity of genomic rearrangements in cancer cells has long been recognized due to cytogenetic studies of chromosomal content of cancer cells (15). Extensive catalogs of recurrent abnormalities in a wide range of tumors have been compiled from cytogenetic studies^1^^,^^2^. These studies revealed that most of tumors contain structural or numerical chromosomal abnormalities or, most commonly, both of them in different proportions. The karyotypic complexity of cancer genomes is reflected in the aneuploidy of cancer cells. Aneuploidy is a state of unbalanced number of chromosomes or large segments of chromosomes. An alteration in the number of whole chromosomes is termed whole-chromosome aneuploidy. Segmental aneuploidy refers to unbalanced regions of chromosomes and is a result of non-reciprocal structural abnormalities.

Extensive studies by chromosomal and array CGH (aCGH) demonstrating frequent alterations in multiple regions of the genome, highlighted the complexity of copy-number changes and the existence of numerous sub-microscopic gains and losses in cancer genomes (16–18). Patterns of copy-number alterations (CNAs) have been associated with cancer type and, sometimes, subtype as well (19). Before the arrival of high-throughput sequencing techniques many cancer-related genes has been already discovered through analysis of individual candidate genes located in the regions of translocations, amplifications, deletions, and LOH in cancer cells.

Studies of hundreds of tumor samples by aCGH demonstrated that a large part of a cancer genome is usually affected by aneuploidy (20). A study of somatic CNAs (SCNAs) by aCGH in 3,131 cancer samples corresponding to 26 histological types found 25% of the cancer genome to be affected by whole arm or whole-chromosome SCNAs, whereas 10% is affected by focal SCNAs. There is a 2% overlap between both types (18). The investigators observed an average of 24 gains and 18 losses per cancer genome as well as a mean of 17 and 16% of the genome gained or deleted, respectively. In many cases, recurrent chromosomal aberrations target known oncogenes or tumor-suppressor genes whose expression levels are altered by the genomic changes.

Perturbations in epigenetic gene regulation that occur in the absence of any change in DNA sequence also alter expression of oncogenes and tumor-suppressor genes (21). Interplay between genetic and epigenetic changes add another level of complexity to studies of cancer genome alterations.

Due to the recent development of next generation sequencing (NGS) and other whole-genome screening methods it is possible to examine the complexity of genomic alterations considering simultaneously point mutations, structural rearrangements, and copy-number changes, gene expression, methylation and their relative contribution into carcinogenesis. For instance, colorectal cancers showed in the majority of samples (84%) high aneuploidy and low mutation level; and in a minority of samples (16%), low aneuploidy and a high mutation level, due to microsatellite instability (MIN) or *POLE* aberrations (22). Endometrial carcinomas displayed a highly aneuploid group with low mutation level (26%) and two groups with low aneuploidy: one with highly elevated mutation level (35%; 7% due to *POLE* aberrations and 28% due to MIN); and surprisingly, a second group with low mutation level (39%), suggesting that perhaps another driving force could be involved (23). CpG island methylation phenotype (CIMP) was associated mainly with MIN group in endometrial carcinomas and colorectal cancers (22, 23). Ovarian carcinomas showed low mutation and high aneuploidy levels almost in all samples analyzed by NGS (24). These data confirmed different patterns of genomic complexity and instability in different types of cancer; as well as the existence of mutation-instability-driven and CIN-driven malignancies.

There is considerable inter-tumor heterogeneity/variability in the degree to which tumor genomes are aberrant at the chromosomal level. Some tumors have only few chromosomal aberrations whereas others may contain dozens. The aberration spectrum differs in tumors that arise in different anatomical sites and in histologically distinct tumors that arise in the same anatomic location (1, 12, 25).

## Intra-Tumor Heterogeneity

In addition to the inter-tumor heterogeneity of genomic rearrangements, cancers display intra-tumor heterogeneity – differences in genomes between malignant cells within the same tumor. By the time of diagnosis, many tumors are composed of heterogeneous populations of tumor cells. In the majority of cancers, the intra-tumor heterogeneity is a result of elevated rates of chromosomal instability and relatively normal rates of point mutations (26).

Application of aCGH and sequencing techniques to diverse tumor types revealed complex subclonal architecture in human cancers (27). The intra-tumor clonal chromosomal heterogeneity of cancers has been known since the 70s (28). The initial studies used G-banding and fluorescence *in situ* hybridization techniques to uncover different intra-tumoral patterns of structural and numerical chromosomal aberrations. Intra-tumor heterogeneity was visualized by the coexistence of cytogenetically related cell populations (sidelines) that share several common chromosome anomalies and exhibit unique karyotypic characteristics. Many observations of intra-tumor heterogeneity of chromosomal aberrations in human cancers exist for squamous cell carcinoma of the skin (29), breast cancers (30–32) gliomas (33), bone and soft-tissue sarcomas (34), pancreatic cancers (35). For instance, 35% of breast cancers and 80% of pancreatic cancers had distinct, but related clones. Moreover, karyotypically unrelated clones were found in 25% of the breast cancers and in 40% of the pancreatic cancers. These unrelated clones were usually near-diploid, carried simple numerical or structural aberrations (sometimes multiple), and were found together with grossly aneuploid, highly abnormal cell population (35). Tumor polyclonality was “rediscovered” recently by cancer genome sequencing based on mutation and chromosomal aberration analysis as well (36).

In addition to clonal chromosomal aberrations cancer cells with CIN display a vast range of random aberrations due to persisting chromosomal instability. Each cell in a cancer cell population could be different from the others because of the presence of non-clonal rearrangements in addition to clonal ones.

The development of single-cell sequencing approaches is another avenue for aiding in our quantitative understanding of intra-tumor cell-to-cell heterogeneity. Characterizing the genomic features of individual cells – rather than a mixed population of tumor cells – helps in resolving the mixtures of genetically distinct cells in a bulk tumor. The first study of so-called single-nucleus sequencing used single nuclei from breast cancers and performed low-coverage sequencing to characterize intra-tumoral DNA copy-number variation (36). Since this study was undertaken, additional studies of single-cell exome sequencing of human tumors (specifically, clear-cell renal cell carcinoma and a myeloproliferative neoplasm) have explored the potential capability of single-cell genomics (37, 38). High levels of clonal and non-clonal genomic heterogeneity were observed in these studies.

What became most evident from the high-throughput comprehensive interrogations of cancer genomes?

(1) The existence of mutation-driven and CIN-driven cancers (majority of analyzed samples) was confirmed;
(2) A high level of inter- and intra-tumor mutational and sub-microscopic structural chromosomal heterogeneity was observed in many types of cancers in combination with large-scale chromosomal heterogeneity;
(3) A process that involves massive *de novo* structural rearrangement called chromothripsis was discovered (39). A key feature of chromothripsis is the formation through most likely a cataclysmic event of tens to hundreds of locally clustered DNA rearrangements.

Genomic heterogeneity together with epigenomic plasticity is translated into phenotypic proteomic heterogeneity of cancer cell populations. Huge collection of coexisting subclones provides opportunities for an endless selection process. These findings suggest also that the linear clonal progression model of cancer evolution, in which cancers progress through single-cell clone bottlenecks, might be an oversimplification. The genetic heterogeneity and the branching evolutionary trajectories evoke a remarkably Darwinian perspective of the evolution of cancer cells. Progression to malignant disease frequently involves diversification and non-linear dynamics of clonal evolution. Metastases often contain genetic aberrations not found in the primary tumor or found in a minor side clone (14, 40) and minor clones provide a reservoir for relapse in one third of multiple myeloma patients (41). Intra-tumor genetic heterogeneity suggests that the therapeutic targeting of cancer-initiating cells is a considerable challenge. It is becoming increasingly evident that intra-tumor genetic heterogeneity is one of the underlying causes of resistance to systemic therapy (42). The immediate consequence of the branching progression model should be improved prioritization of therapy targets and search for “founder” events. The urgent need to address the risk of misleading conclusions based on single biopsies is also apparent (43).

## Chromosomal Instability as a Type of Genomic Instability

Genomic heterogeneity is a consequence of genomic instability of cancer cells. Genomic instability refers to increased rates of alterations in the genome during the life cycle of cells.

Based on the scale of genomic changes there are several distinct forms of intrinsic genomic instability: nucleotide instability due to frequent mutations (NIN), MIN, structural genomic/chromosomal instability (S-CIN), and W-CIN. These four types of instability are not mutually exclusive, and can coexist in the same cells, with S- and W-CIN being the most frequently found in cancer forms of instability. S- and W-CIN overlap, but for the most part are independent (44).

The focus of this review is W-CIN – increased rates of chromosomal mis-segregations relative to normal diploid cells. The term CIN became widely used since it was introduced by the groundbreaking work of Lengauer et al. (45), but very often it is used instead of aneuploidy, or karyotypic complexity, or heterogeneity. Actually, CIN means the presence of instability in cancer cells in the present or in the past, and the main characteristic of CIN instability is a rate of instability. For instance, S-CIN is equal to a number of acquired structural rearrangements per cell per division. Similarly, W-CIN can be measured by the amount of gained or lost chromosomes per cell per division. W-CIN, which consists of elevated rates of chromosome mis-segregation, must be distinguished from aneuploidy, which is a state of abnormal chromosome number. Defining CIN as a process where rates may vary led to the realization, that ongoing instability represents persistent defects in equal distribution of chromosomal material into daughter cells during cancer cell propagation (45, 46).

The presence of chromosomal alterations in a tumor does not necessarily indicate that the instability persists in the tumor. Detection of structural and numerical chromosomal alterations and, especially, heterogeneity of these alterations indicate that instability occurred in the past, but the question of ongoing instability usually requires additional investigation. The level of W-CIN cannot be determined by simply scoring of genomic alterations, but requires scoring of mitotic abnormalities, including lagging chromosomes and multipolar mitoses, as well as gains and losses of chromosomes per cell per division (47). On average, W-CIN colorectal cancer cells mis-segregate a chromosome once in every one to five cell divisions (45, 48). A wider range of W-CIN rates was detected in ovarian, lung, melanoma cancer cells: between one chromosome lost or gained in 20 cell divisions in ovarian cancer cell lines up to several chromosomes lost or gained per cell division in malignant melanoma and lung cancer cell lines [(49) and unpublished data].

## Molecular Mechanisms Promoting W-CIN

The precise molecular mechanisms that may cause CIN in tumor cells just recently became more evident. Studies aimed at identifying the mitotic defects that may be responsible for inducing chromosome mis-segregation in cancer cells (W-CIN) show that these defects include spindle assembly checkpoint (SAC) dysfunction, kinetochore attachment errors, mitotic spindle defects, and other cell division inaccuracies (50–60). There are many molecular defects leading to chromosomal mis-segregation in model systems, but which of them are present in cancer cells? Large-scale genome sequencing has revealed very few mutations in genes that encode proteins involved in chromosome segregation during mitosis (22, 24, 26, 61), but reiterated that cancer-causing mutated genes encode proteins involved in cell cycle control and cell signaling pathways responsible for cell growth and death. Formerly, the persistent mis-segregation of chromosomes in tumor cells has been largely attributed to errors arising during mitosis that were not directly linked to the driver mutations in oncogenic signaling pathways. Emerging data show that CIN and the oncogenic signaling pathways responsible for driving tumor formation are closely interrelated [reviewed in Ref. (62)].

### Defects in mitotic-checkpoint signaling

A weakened mitotic-checkpoint may allow cells to enter anaphase in the presence of unattached or misaligned chromosomes. An extensive search for mitotic-checkpoint defects in human cancers has uncovered very infrequent mutations of mitotic-checkpoint genes (63) and more frequent altered expression of mitotic-checkpoints genes BUB1, BUBR1, BUB3, MAD1, MAD2 [reviewed in Ref. (64)].

Mitotic-checkpoint dysfunction has been extensively studied in mouse models [reviewed in Ref. (65–67)]. In all cases, mice with genetically reduced levels of mitotic-checkpoint components have an increased level of aneuploidy and CIN in mouse embryonic fibroblasts (MEFs) and tissues. Partial loss of SAC function is responsible for causing W-CIN. Evidence in favor of this view is derived from the high incidence of aneuploidy and tumorigenesis in mice engineered to have weakened SAC activity (68, 69). Moreover, in humans, reduced SAC activity has been observed in individuals with Mosaic Variegated Aneuploidy (MVA), an extremely rare disease strongly linked to mutations in SAC component BubR1 (70). Although aneuploid animals with reduced levels of BUB1, BUBR1, BUB3, RAE1, or both RAE1 and NUP98 fail to display an increase in spontaneous tumorigenesis, these mice are prone to carcinogen-induced tumors (69, 71–73), suggesting that aneuploidy does not initiate cancer in these mouse models, but rather drives tumor formation in cases in which mutations at oncogenic or tumor-suppressor loci have already increased the potential for cellular transformation.

### Merotelic attachment

One kinetochore can attach to microtubules from both poles of the spindle and form a merotelic attachment. Kinetochores in human cells bind approximately 25 microtubules and errors in the orientation of kinetochore-microtubules attachments arise through the stochastic nature of interactions between microtubules and kinetochores. If these attachments persist into anaphase then lagging chromatid pairs might be mis-segregated or excluded from both daughter cells during cytokinesis. Unlike other mal-orientations, merotely evades SAC detection (74) since kinetochores attain full occupancy of microtubules (with improper orientation).

Cancer cells with CIN have excessive rates of formation of merotelic attachments (52, 53) and diminished capacity to correct them (48, 75–77). It was shown that relatively minor perturbations in kinetochore-microtubule attachment dynamics are sufficient to disturb attachment stability required for faithful chromosome segregation, and restoration of kinetochore-microtubule attachment dynamics leads to the suppression of W-CIN (48, 75–77). Persistence of errors in kinetochore-microtubule attachments was revealed by live imaging of cancer cells (48). Direct measurements show that many W-CIN cancer cells have hyperstable kinetochore-microtubule attachments which undermine their ability to correct errors and leads to high rates of chromosome mis-segregation. Importantly, increasing the detachment rate of kinetochore-microtubule improves error correction and is sufficient to restore faithful chromosome segregation in W-CIN cancer cells (58, 62, 75, 76).

### Multiple centrosomes

Cells that possess more than two centrosomes might form multiple spindle poles during mitosis. If this defect is not corrected then a multipolar division might occur, resulting in the production of highly aneuploid and often non-viable daughter cells. Increased centrosome number correlates with aneuploidy and it has been well documented that cancer cells frequently enter mitosis with more than two centrosomes leading to multipolar spindles [reviewed in Ref. (59, 77, 78)]. However, centrosomes in multipolar spindles often cluster into two groups to allow cells to divide in a bipolar fashion (79–81). Centrosome clustering may increase the frequency of incorrect kinetochore-microtubule attachments [such as merotelic attachments (see previous section)]. Extra centrosomes are therefore capable of driving chromosome mis-segregation through a mechanism that is independent of multipolar divisions. Additional support comes from the realization that transient defects in spindle geometry in cancer cells, such as those caused by supernumerary centrosomes, elevate the incidence of merotelic attachments, indicating that some cancer cells with CIN have excessive rates of formation of attachment errors (52, 53). Thus, extra centrosomes increase the rate of formation of kinetochore-microtubule attachment errors leading to W-CIN.

### Cohesion defects

The integrity of centromeric structure ensures that kinetochores are positioned in a back-to-back configuration. If sister chromatid cohesion is lost prematurely or persists during anaphase, chromosomes can be mis-segregated. It has been shown that defects in pericentromeric cohesion undermine the establishment of proper kinetochore-microtubule attachments (82). The consequence is an increased rate of formation of kinetochore-microtubule attachment defects that leads to elevated rates of chromosome mis-segregation.

When genes that have putative functions in guarding against chromosome mis-segregation were systematically sequenced in a panel of aneuploid colorectal cancers (63), 10 of the 11 mutations identified were in genes that directly contribute to sister chromatid cohesion, indicating that defects in the machinery that controls sister chromatid cohesion might promote aneuploidy. However, those studies only explain the cause of W-CIN in a minority of tumor cells. Consistently, overexpression of separase or securin, two key regulators that control the loss of chromatid cohesion, promotes aneuploidy and cellular transformation (83, 84).

### Oncogene-induced mitotic stress model

A recently proposed oncogene-induced mitotic stress model explains how most tumors can become aneuploid in the absence of mutations in the mitotic-checkpoint genes (85). The explanation is that activated oncogenes affect also the mitotic process that controls chromosome segregation. Emerging data show that W-CIN and the oncogenic signaling pathways responsible for driving tumor formation are closely interrelated, and novel roles for oncogenes and tumor-suppressor genes in genome stability are proposed [reviewed in Ref. (62, 86)]. These include genes participating in the RB pathway, the APC pathway, the WNT signaling pathway, the Ras signaling pathway, the TGF-beta signaling pathway, the NF-kB pathway, integrin signaling and cell adhesion, the Hippo signaling pathway, and the DNA damage response. Originally, many of these genes were thought to be tumor-suppressive or oncogenic solely because of their role in proliferative control. But the oncogenic signaling pathways seem to play dual roles: they act as drivers for tumorigenesis and they induce W-CIN. This connection to W-CIN arises from the disruption of the careful orchestration of events required for accurate chromosome segregation during mitosis. This entails decreasing the rate of correction of kinetochore-microtubule attachment errors and/or increasing the rate of formation of those errors through extra centrosomes or by disrupting the centromere geometry (62). The notion that tumor-suppressor genes (and by extension, oncogenes) combine their known roles in cell cycle progression, growth and differentiation with the induction of genomic instability has a substantial body of evidence to support it [reviewed in Ref. (86)]. Because of the frequency with which they are disrupted in cancer, chromosome instability caused by their dysfunction may be more central to tumorigenesis than previously thought (62).

Another recent proposition about how W-CIN can arise is that replication stress causes whole-chromosome mis-segregation by premitotic events, with structural chromosome abnormalities precipitating chromosome mis-segregation in mitosis (87). DNA replication stress has been observed across several tumor types (88), and in the light of recent findings there is a need to search for the mechanism that could cause whole-chromosome mis-segregation as a consequence of replication stress independently of defects of the segregation process in mitosis.

## Consequences of CIN

### Aneuploidy

Aneuploidy is a direct consequence of W-CIN. We distinguish two types of aneuploidy in cells with W-CIN: random and clonal. Since chromosomes are mis-segregated during cell divisions this results in random aneuploidies. When random aneuploidy provides proliferation advantages and is selected for, it becomes clonal (selected) aneuploidy.

Clonal aneuploidy could be low-grade, with only one or few chromosomal disbalances and near-diploid genome, and high-grade or gross aneuploidy with many clonal chromosomal gains and losses that produce extremely disbalanced genomes, usually with elevated ploidy levels as well. Deviation from low-grade aneuploidy to high-grade aneuploidy requires overcoming limitations exerted by the p53 pathway (48). These limitations seem to be also relevant to the acquisition of ploidy instability and polyploidization of low-grade-aneuploid/near-diploid genome that allows cells to propagate with very elevated rates of W-CIN, mainly due to the “buffering” effect of higher chromosome number (89).

### Heterogeneity of karyotypes

Heterogeneity of chromosomal contents of cells in a cell population with W-CIN is another direct consequence of random unequal distribution of chromosomes during mitosis. The presence of W-CIN results in wide spectrum of karyotypes with gains and losses of different chromosomes in different combinations. We distinguish clonal heterogeneity and cell-to-cell heterogeneity. Clonal heterogeneity – a coexistence of several related clones in a cellular population, is a result of chromosomal instability in the past with further selection of these clones. Cell-to-cell heterogeneity is related to random gains and losses of chromosomes in cells and is an indicator of the presence of ongoing chromosomal instability.

Studying phenotypic switching due to changes of chromosomal content in glioblastoma, Gao et al. (90) came to the conclusion that chromosomal instability generates genomic diversity in the tumor cell populations (and therefore transcriptome diversity), to allow for environment-facilitated clonal expansion. Studies in microorganisms show that heterogeneity in the DNA could be beneficial in certain circumstances, increasing survival under selection pressure (91, 92).

### W-CIN promotes DNA damage and genomic instability

It has been shown that chromosome segregation defects can lead to DNA damage. For example, lagging chromosomes in anaphase tend to be trapped in the cytokinetic furrow resulting in DNA double strand breaks (93, 94). Also, lagging chromosomes tend to form micronuclei in the subsequent G1 phase. The chromosomes trapped in micronuclei do not replicate on time with the major nucleus, are prone to having defects in DNA replication which cause additional genomic instability, and could even be occasionally pulverized in the subsequent mitosis leading to chromothripsis (95). These results reveal a potentially vicious cycle. Chromosome segregation errors lead to DNA damage and that damage may promote further chromosome mis-segregation and other forms of genomic instability (20). It was suggested that W-CIN promotes itself because imbalances due to aneuploidy further destabilizes symmetrical chromosome segregation (96).

Aneuploid yeast strains also exhibited increases in genomic instability with elevated rates of point mutations, mitotic recombination, and further loss of whole chromosomes, as well as defective DNA repair (97).

## Cellular and Organismal Responses to W-CIN and Aneuploidy

Changes in copy-number of a chromosome can lead to phenotypes associated with copy-number changes of a specific gene or several genes located on this chromosome, as well as to general effects due to changes in copy-number of numerous genes. Whole copy-number chromosomal alterations promoted by CIN come with certain costs and benefits for the propagation and survival of the cell. Large-scale changes in DNA copy-number can cause detrimental phenotypes due to the cumulative effects of CNAs in many genes simultaneously (98). First of all, these changes have adverse effects on fitness. The most illuminating data came from the work on yeast strains. Torres et al. (92) used a chromosome transfer strategy and selectable markers to generate aneuploid yeast strains with a single extra chromosome. The aneuploid yeast proliferated more slowly than the wild-type cells, demonstrated delay in the G1 phase of the cell cycle as well as increased sensitivity to drugs targeting protein synthesis and folding, and metabolic changes with increased glucose uptake (97). Similar to disomic yeast strains, trisomic MEFs show signs of energy and proteomic stress (99, 100).

Lately, stress/slow-growth-related transcriptional signature present in aneuploid cells was identified for diverse organisms and was largely independent of the identity of the extra chromosome (101). These results demonstrated that random aneuploidies of different chromosomes and in different organisms impact similar cellular pathways and cause a stereotypical antiproliferative response: genes that were involved in the response to stress were consistently upregulated, and genes associated with the cell cycle and cell proliferation were down-regulated in aneuploid cells.

Gene copy-number changes are generally translated into changes in gene expression (102). Quantitative proteomic analyses in budding yeast and human cells showed that copy-number changes result in changes in protein production in many cases (103, 104) with the exception of proteins that are predominantly components of large protein complexes, presumably, because unassembled units of such complexes are generally unstable. For instance, stoichiometry of ribosomal complex is maintained by the proteolysis of subunits that fail to assemble into the complex (105). It was proposed, that aneuploidy could lead to proteotoxic stress due to accumulation of unfolded, misfolded, aggregated proteins in a cell, and can lead to the activation of ubiquitin-proteasome and chaperone pathways activation which, in turn, could require additional energetic resources (106).

Cells with W-CIN, similar to aneuploid, also have proliferation defects and show signs of cellular stresses (48). In addition, high level of chromosome mis-segregation cause activation of p53, which results in G1 arrest and apoptosis (54), whereas lower levels of chromosome mis-segregation induce a p53-mediated cellular arrest (107). It was suggested, that chromosome mis-segregation leads to lagging chromosomes and anaphase bridges, which become damaged during cytokinesis and trigger DNA damage response. Therefore intact DNA damage response limits the development of W-CIN. Effects of W-CIN are summarized in Table 1.

**Table 1:** **Defects leading to CIN, effects of CIN, and adaptations to chromosomal instability**.

Defects leading to W-CIN	Mitotic-checkpoint relaxation
	Centrosome overduplication
	Cohesion defects
	Merotelic attachment
Effects of W-CIN	Random aneuploidy
	Karyotypic heterogeneity – basis for phenotypic selection
	Loss of heterozygosity
	Loss of tumor-suppressors
	Gain of oncogenes
	Selected, clonal aneuploidy
	Slow proliferation
	DNA damage, genomic instability
	Unfit cells
	Proteotoxic stress
	Increased energy needs
	Occasional changes of genome favorable for cell survival and proliferation
Adaptations
Cellular	Centrosome clustering
	Protein-level dosage compensation
	Changes in proteasome-mediated protein degradation pathway
	ATM-p53 pathway inactivation
	Polyploidy
Tissue/organ/whole organism	Response to tissue damage, inflammation, tissue remodeling

## Adaptations to W-CIN and Aneuploidy

Chromosome mis-segregation compromises the proliferation of cells, indicating that additional changes in combination with elevated chromosome mis-segregation rates are required to generate grossly aneuploid cells (48).

How cells can adapt to tolerate detrimental effects of chromosomal instability and aneuploidy? Since propagation of diploid human cells with mis-segregation of chromosomes is limited by activation of the p53 pathway (54), deletions or mutations of the p53 gene are required to allow propagation of cells with W-CIN. This notion fits with the observation, that p53 pathway is one of the most frequently disrupted pathways in many types of cancer (108). Loss of p53 allows highly aneuploid cells to proliferate *in vitro* (54, 94, 107), but it does not directly cause euploid cells to become aneuploid. This point is further supported by data showing that knockout of p53 alone does not lead to chromosomal gains/losses (109), therefore, p53 does not play a causative role in the creation of instability, but its role is rather permissive/adaptive.

Another adaptive mechanism to mis-segregation of chromosomes is a switch to a genome with a higher level of ploidy that tolerates multiple chromosomal gains and losses (110). For instance, tetraploid yeast can tolerate almost 1,000-fold increase in the rate of chromosomal gains and losses without impairment of the cell cycle progression (111). Diploid yeast strains with an extra chromosome are far less sensitive to drugs that target protein synthesis and protein folding than isogenic haploid strains with the same chromosome gain (92). Similarly, tetraploid mammalian cells with CIN and aneuploidy have a near-normal growth rate compared with isogenic diploid cells (52). Among the cancer cell lines from the NCI-60 panel, the most heterogeneous and chromosomally unstable cell lines have grossly aneuploid karyotypes in near-tetraploid/near-pentaploid range [(44) and unpublished data]. In addition, ploidy instability and the presence of major or side clones with high ploidy levels was detected in the majority of grossly aneuploid cancer cell lines from this panel (44). Clearly, there is a connection between high chromosomal instability rates and polyploidization, pointing to an adaptive role of this process in acquisition of high rates of W-CIN allowing further selection of a genome with gross aneuploidy.

Another characteristic feature of W-CIN cancer cells is the presence of supernumerary centrosomes. The biological consequence of this could be a multipolar spindle, which generally is antagonistic to cell viability and proliferation. Although centrosome amplification has been linked to aneuploidy and multipolar spindles, there is an observation that extra centrosomes usually cluster together in two groups during mitosis to prevent spindle multipolarity (79–81). This centrosome clustering represents another mechanism of cellular adaptation to W-CIN-promoting conditions.

Several adaptive responses to random aneuploidy were dissected from the work on a panel of aneuploid yeast strains with different additional chromosomes. Most of these strains grew slowly, exhibited a delay in G1 phase, of the cell cycle and demonstrated increased metabolic requirements (92). It was proposed that aneuploidy-induced stoichiometric imbalances in proteins might severely stress proteasomes (91, 97). Interestingly, after aneuploid strains were grown continuously for 14 days, genetic alterations that improved their proliferative potential were then identified by whole-genome sequencing, and two strains independently acquired loss-of-function mutation of ubiquitin-specific protease UBP6 (112). Deletion of UBP6 was found to improve the growth rates of four disomic strains, and quantitative mass spectrometry demonstrated that loss of UBP6 led to attenuation of the level of proteins overproduced due to random aneuploidies (97, 112). Such mutations represent an adaptive response to adverse effects of random aneuploidies. Since aneuploidy lead to increased mutational rate and structural genome instability, acquisition of adaptive changes might be accelerated by aneuploidy-induced genomic instability (113).

Adaptations to W-CIN might occur not only on a cellular level, but on tissue and organismal levels as well (114). For instance, prolonged DNA damages signaling, a consequence of W-CIN, leads to secretion of inflammatory cytokines. Moreover, as a result of excessive W-CIN, some cells can trigger inflammatory response that promotes tumorigenesis (115). Inflammation and tissue remodeling can facilitate tumor progression due to the production of growth factors, cytokines, chemokines, prostaglandins, and angiogenic factors, leading to further W-CIN tumor adaptations in the context of a response to a tissue damage physiological program (116). Table 1 summarizes possible adaptations that allow cells with W-CIN to survive.

## W-CIN, Aneuploidy, and Cancer: A Complex Relationship

Since first experimental findings showed that chromosome mis-segregation (W-CIN) and the following aneuploidy can promote or inhibit tumorigenesis, several approaches have been taken further to examine the impact of aneuploidy and W-CIN on tumorigenesis [reviewed in Ref. (117–120)]. Studying tumorigenic effects of aneuploidy *per se* is limited by the existence of only few aneuploidies viable in mammals: two constitutional trisomies can survive infancy in humans, and none can survive embryonic development in mouse. Individuals with trisomy 21 have an increased risk for acute myeloid leukemia, but decreased risk of developing solid tumors. It seems that Down and Edwards syndromes increase the risk of developing childhood cancers (120). This increased risk was explained by chromosome-specific effects, but not by the impact of random aneuploidy (121). Women with Turner syndrome have an increased risk of developing gonadoblastoma and brain tumors (122). Is chromosomal instability present in cells from individuals with constitutional trisomies? Using FISH probes for chromosomes 8, 15, and 18, Reish et al. (123), showed that elevated number of random aneuploidies is present in the lymphocytes of individuals with Down, Edwards, and Patau syndromes, as well as Turner syndrome. Therefore, existing data suggest that trisomic cells from patients from Down, Edwards, Patau, and Turner syndromes have an elevated level of W-CIN compared to cells from normal diploid individuals. If elevated cancer susceptibility can be attributed to W-CIN or specific aneuploidies present in the cells of these trisomic patients, remains to be elucidated. Involvement of W-CIN is supported by the fact that the only known human heritable syndrome with numerical chromosomal instability due to biallelic loss-of-function mutation in the spindle checkpoint component BubR1, MVA, clearly predispose to cancer.

To understand the role of W-CIN in tumorigenesis, mouse models with decreased fidelity of chromosome segregation have been generated. Such models of W-CIN use genetic alterations that interfere with either chromosome segregation machinery or with SAC function. Results of these studies have been summarized in several reviews (114, 118, 121) and will not be discussed in details here. The studies using mouse models have shown that W-CIN may cause or accelerate tumorigenesis more readily in some organs than in others; and introduction of W-CIN into mouse models of cancer has tumor-promoting and tumor-suppressive effects. These studies also show that both weakening and hyperactivating of SAC is sufficient to promote W-CIN and to induce tumorigenesis. Moreover, crossing mice with mutated SAC components into mice homozygous for p53 deletion, increased tumorigenesis and decreased survival compared with either mutation alone (107). Thus, in the absence of p53 that limits the proliferation of chromosomally unstable cells, more aggressive disease is consistently observed.

## Cellular Functions and Processes Associated with W-CIN in Cancer Cells

Relationships between W-CIN and cellular functions of cancer cells require further investigation. Specific cellular processes have been associated with chromosomal heterogeneity in cancer cells (124). It has been found, that cell-to-cell heterogeneity is a good substitute for W-CIN [(47) and our unpublished data].

Distribution of genes whose expression correlated with the level of chromosomal heterogeneity indicated that cell communication and signal transduction, cell adhesion, motility, and migration, response to wounding and inflammatory response, negative regulation of cell proliferation, and DNA replication are the main biological processes associated with the level of heterogeneity of chromosomal content in the cancer cells. Moreover, genes correlated with chromosomal heterogeneity fell into two groups based on their positive or negative correlation coefficients, showing a striking difference between them. Genes, whose expression positively correlated with the higher levels of chromosomal heterogeneity, fell into GO categories such as cell communication and signal transduction, including cell surface receptor-linked signal transduction, cell adhesion, locomotion, motility, and migration, development, morphogenesis, and differentiation, response to wounding, and inflammatory response. Genes, whose higher expression negatively correlated with the high levels of chromosomal heterogeneity, fell into totally different GO categories: cellular metabolism, nucleic acid metabolism, regulation of transcription, DNA replication, response to DNA damage stimulus, DNA repair, chromosome organization and biogenesis, DNA packaging, chromatin condensation, unwinding and replication initiation, and base-excision repair, cell cycle regulation (124). This pattern of gene signatures is consistent with meta-pathway “tissue remodeling” (125) for cancer cells with higher levels of chromosomal heterogeneity; and with meta-pathway “proliferation” for cancer cells with lower levels of chromosomal heterogeneity.

The lower expression of genes involved in DNA damage checkpoints (CHK1, CHK2, H2AX, RAD21, XRCC5, DDB1) and DNA replication prevention (BCCIP, BRCA2, CDT1, MCM2-7, cyclin B2) correlated with higher numerical chromosomal heterogeneity. The expression levels of genes involved in DNA packaging, chromosome condensation, and kinetochore formation (H3 histone, H1FX, H2AX, H2AZ, TOP1, RCC1, RCC2, SMARCA5, RCBTB1, CENPC1, ZWINT) are also relatively down-regulated in cancer cells with higher levels of chromosomal heterogeneity compared to cancer cells with a lower levels of heterogeneity.

A collective molecular portrait associated with chromosomal instability in cancer cells includes relative up-regulation of genes that are associated with increased motility and migration, epithelial-mesenchymal transition (EMT), and are critical for tumor invasion and metastasis: RhoC, fibronectin, LOX, TWIST, SNAI2, EGFR, laminins, integrins, collagens, CDC42 effector protein (Rho GTPase binding), Rho family GTPase 3, RAB, CXCL2, TGF-b2, VEGFC, IL-6, IL-8, CTGF, vimentin, N-cadherin, CD44, BCAR3, protocadherins, MMP2 and MMP14, NOTCH2, SERPINE1, 2, and 8, IGFBP3 and 7, TNFAIP3, TNFRSF12A and 19, PLAUR, and SPARC (124).

## Cancer Chromosomal Instability Adaptive Phenotype

The presence of chromosomal instability in cancer cells produces a multi-layered phenotype which comprises an increased predisposition to chromosome mis-segregation during mitosis; an aberrant repair of DNA breaks, and a survival state specifically adapted to aneuploidy and to the constant reshuffling of the genome (126). Identifying molecular, cellular, and microenvironmental processes underlying and promoting this complex W-CIN phenotype will be a key step toward understanding cancer development in general and drug resistance in particular.

**Figure 1:**
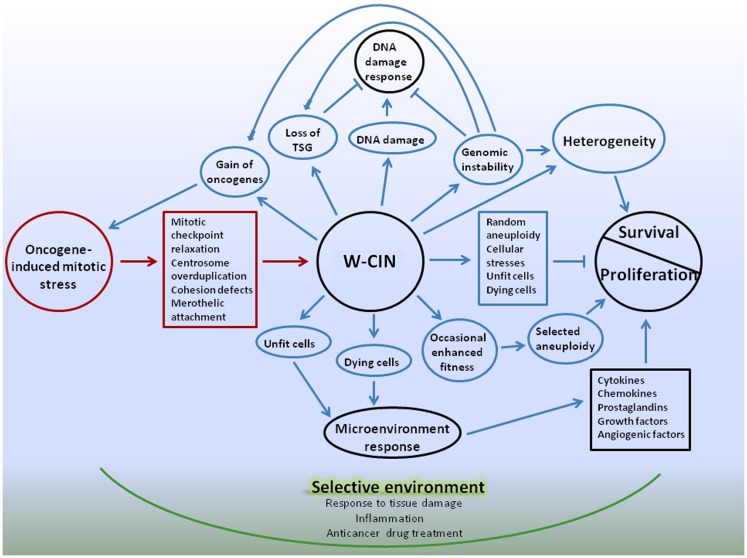
**Schematic representation of W-CIN adaptive phenotype model**. Oncogene-induced mitotic stress promote mitotic-checkpoint relaxation, overduplication of centrosomes, defects in chromatid cohesion, and merotelic attachments of microtubules to kinetochores, leading to persistent chromosome mis-segregation during mitosis – W-CIN. Direct consequences of W-CIN are mainly disadvantageous for cell proliferation due to genomic imbalances produced by random aneuploidy, but beneficial for survival due to heterogeneity of cellular phenotypes. Proliferation of aneuploid cells is slower than their normal counterparts because cellular fitness is decreased due to proteomic, energy, and other stresses. At the same time heterogeneity allows cells to survive in changing microenvironment, and occasional genomic changes that produce enhanced fitness of phenotypes can be selected, giving cells proliferative advantages. Indirect consequences of W-CIN can be both advantageous and disadvantageous for proliferation of cells, but, again, beneficial for survival. Due to DNA damage and genomic instability induced by W-CIN, additional random genomic changes occur. These changes could give cells proliferative advantages or disadvantages, or be neutral. DNA damage response protects cells from development of CIN, unless cells acquired defects in the DNA damage checkpoint that allow them to proceed through cell cycle and avoid apoptosis. Acquisition of defects in the DNA damage checkpoint can be facilitated by occasional favorable genomic changes in cells with W-CIN. At the same time, additional heterogeneity of cellular phenotypes due to genomic instability enhances chances of cellular population with W-CIN to sample microenvironment and survive. Due to phenotypic heterogeneity of the fitness of CIN cells, and the presence of unfit and dying cells in the cell population, microenvironmental response to tissue damage can contribute to proliferation and survival of W-CIN cells. For instance, response to tissue damage can provide support for the proliferation of better fitted W-CIN cells; enhance their chances for survival and acquisition of new adaptations through genomic/phenotypic changes. These changes are going to be selected in the microenvironment, shaped by physiological responses to tissue damage. Adaptive interactions with microenvironment can provide CIN cells with opportunities for gradual internalization of proliferative and survival signals. Anticancer drugs also create selective microenvironments and promote selection of CIN phenotypes suited to survive applied treatments. Targeting W-CIN cancer cells requires taking into account multiple layers of W-CIN adaptive phenotype. Color coding: red – causes of W-CIN; blue – consequences of W-CIN.

Following is a summary of the complex effects of W-CIN on proliferation and survival of cellular populations, and an introduction of a possible model for a cancer multi-layered W-CIN adaptive phenotype (Figure 1).

Understanding how W-CIN promotes adaptations can be aided by the concept of Darwinian selection, which depends on cell-to cell variations with survival of the fittest in a selective environment (127). Adaptation is a characteristic feature of human neoplasms; and the ability of tumors to adapt to external pressures comes from tumor cell chromosomal heterogeneity – a direct consequence of W-CIN. Besides heterogeneity, another direct consequence of W-CIN is random aneuploidy. Aneuploidy invokes multiple cellular stresses and is detrimental for proliferation of cells, unless occasional beneficial changes are acquired. Therefore, an array of chromosomal gains and losses produced by W-CIN slow tumor progression due to the detrimental consequences of genomic disbalances, but eventually can drive tumor progression by accelerating the gain of oncogenic and the loss of tumor-suppressor loci, and promoting acquisition of cellular adaptations to genomic disbalances.

Since whole-chromosome mis-segregation accelerate further chromosomal instability, genomic damage, and acquisition of mutations, cells can occasionally obtain features that are beneficial for proliferation and survival, facilitating tumor progression. However, the random nature of chromosomal mis-segregation implies that it can equally promote the loss of beneficial features, for instance, due to loss of oncogenes or gain of tumor-suppressors (128). Due to the heterogeneity of cellular phenotypes in a population of cells with W-CIN, the entire range of different cellular fitness phenotypes could exist in a population. This implies simultaneous coexistence of unfit, dying cells as well as better fitted cells within the population of W-CIN cancer cells. Fitness heterogeneity also may have consequences for proliferation and survival of chromosomally unstable cells because of the response of tissue microenvironment (Figure 1).

Connection between inflammation and genomic instability has been already established due to many observations suggesting that common cellular and molecular mechanisms are active in wounds and in cancer sites. It was suggested that tumors, in particular, carcinomas, activate the latent wound-healing program on the host but in an exaggerated and prolonged manner (129). Most of the genes that orchestrate wound-healing process are important regulators of cancer growth and progression. Chronic inflammation is associated with an increase in cytokines, chemokines, reactive oxygen and nitrogen species, that promote genomic instability (115, 130). In turn, W-CIN may lead to the tumor cells secreting factors that recruit inflammatory immune cells or promote tissue remodeling, including tumor angiogenesis (114).

Chromosomal instability is a multi-layered phenotype that has to be evaluated based on systemic biological approach which includes not only studies of genome, cell cycle, and genomic evolution of CIN cancer cells, but also evaluations of their cellular functions and interactions with surrounding cells and tissues. Existing data suggest that the CIN phenotype is associated with the following features:

(a) Changes in the cell cycle organization and coordination leading to random unequal distribution of genetic material in daughter cells;
(b) Changes in metabolism and cellular functions due to genomic imbalances and alterations;
(c) Intracellular mechanisms of adaptation to the consequences of genome reshuffling;
(d) Extracellular mechanisms of adaptation leading to survival and selection of karyotypically unstable and aneuploid cells as part of a program of the response to tissue damage with gradual internalization of proliferative and survival signals (116).

## Targeting CIN Phenotype

Both the causes and the consequences of W-CIN, as well as the adaptive mechanisms alleviating the detrimental consequences, offer opportunities for therapeutic intervention. For instance, drugs that induce energy stress or inhibit autophagy or protein folding have been shown to specifically inhibit proliferation of trisomic MEFs and aneuploid human cells (99). Similarly, the adaptations to mitotic defects could be targeted. For example, drugs that interfere with centrosome clustering mechanisms could potentially be lethal to tumor cells with multiple centrosomes, but spare normal cells. A genome-wide RNAi screen in near-tetraploid *Drosophila* S2 cells identified a number of genes required for centrosome clustering (131). The classes of genes identified in this screen enabled the identification of a range of cellular processes that control organization of multipolar centrosomes: the SAC components Mad2, BubR1 (human Bub1), CENP-Meta (human CENP-E) as well as genes involved in cell polarity, actin regulation, and cell adhesion.

Taking advantage of the tumor-specific phenotype of centrosomal clustering, a cell-based screening strategy was used to identify small molecules that inhibit centrosomal clustering and thus force tumor cells with supernumerary centrosomes to undergo multipolar mitoses, and subsequently, apoptosis. Screening of a relatively small but diverse natural product extract library led to the identification of griseofulvin, which induced multipolar spindles by inhibition of centrosome coalescence, mitotic arrest, and subsequent cell death in tumor cell lines but not in diploid fibroblasts and keratinocytes with normal centrosome content (132). Following this work, 34 griseofulvin analogs were synthesized and tested as inhibitors of centrosomal clustering (133). The most active analogs were found with a 25-fold increase of activity compared to griseofulvin.

There are multiple possibilities for targeting cancer cells based on the W-CIN phenotype they posses. Interrogation of the data-rich drug discovery panel of the NCI-60 cancer cell lines, used by the National Cancer Institute to screen compounds for anticancer activity, provided a list of potential anticancer agents targeting aneuploid and chromosomally unstable cancer cells (134–136), and confirmed that it is possible to discover potential anticancer agents based on association of their activity with a determinant of karyotypic state. It identified 13 classes of chemical compounds that express more growth-inhibitory activity toward cancer cell lines with more complex and/or unstable karyotypes. These compounds represent a mostly unexplored set of chemical motifs whose activities correlate with the variability of the cellular karyotypes. These results also suggest that the mechanisms of action of many well-known anticancer agents are most likely not associated with aneuploidy and the chromosomally unstable status of cancer cells. Compounds identified in this study may target genes or pathways; however, it is important to recognize that certain agents may be active against the “state” of complexity or instability itself rather than against any specific gene product or pathway.

The possible influence of karyotypic complexity and genetic instability on the response to individual cytotoxic agents has also been recognized. For example, taxanes seemed to have an increased activity in tumors with chromosomal stability; and anthracyclines and platinum agents may target more karyotypically complex tumors. Based on these data, the need for new combinatorial strategies that would target CIN has been suggested (137).

Since survival of W-CIN cancer cells requires extracellular mechanisms of adaptation, genes that are involved in the shutdown of tissue repair response could represent promising targets for cancer therapy (129).

## Conclusion

As discussed above, W-CIN is a widespread feature of many types of cancer. It has both tumor-promoting and tumor-suppressive effects, and their balance could be beneficial or detrimental for carcinogenesis. Chromosomal instability is a multi-layered phenotype that has to be evaluated based on systemic biological approach which includes not only studies of genome, cell cycle, and genomic evolution of W-CIN cancer cells, but also evaluations of their cellular functions and interactions with surrounding cells and tissues. W-CIN mediates evolution of the cancer cell population under selective pressure and facilitates further accumulation of genetic changes that promote malignancy. Due to gross genomic imbalances, W-CIN induces changes in metabolism and cellular functions of cancer cells, as well as intracellular adaptations to the consequences of genome reshuffling. Moreover, CIN could be accompanied by extracellular mechanisms of adaptation leading to survival of karyotypically unstable cancer cell population. These possibilities need to be further explored. The concept of W-CIN multi-layered phenotype can aid in developing new strategies for targeting cancer.

## Conflict of Interest Statement

The authors declare that the research was conducted in the absence of any commercial or financial relationships that could be construed as a potential conflict of interest.

## Footnotes

^1^http://cgap.nci.nih.gov/Chromosomes/Mitelman

^2^http://www.ncbi.nlm.nih.gov/sky/skyweb.cgi
